# The Spatial Non-Equilibrium and Convergence of Chinese Grain Enterprises’ Total Factor Productivity—Evidence from China

**DOI:** 10.3390/foods11182843

**Published:** 2022-09-14

**Authors:** Qinqin Fan, Yangyang Zheng, Wei Jia

**Affiliations:** 1 Institute of Agricultural Economics and Development, Chinese Academy of Agricultural Sciences, Beijing 100081, China; 2 School of Economics and Management, China Agricultural University, Beijing 100083, China; 3 Business School, Wenzhou University, Wenzhou 325035, China

**Keywords:** meta-frontier Malmquist index, total factor productivity, technical efficiency, grain processing enterprises, China

## Abstract

The improvement of grain processing capacity is crucial to the realization of grain security. Enterprises are important grain processing bodies and their productivity directly determines grain processing capacity. Chinese grain processing enterprises still have difficulties, and how to further improve grain processing capacity and the total factor productivity of grain processing enterprises may be an important aspect. We used the meta-frontier Malmquist index to measure the total factor productivity of grain enterprises as well as judge the change trend and regional gap, applying the classical regression model to test the convergence of China’s overall and regional grain enterprises’ total factor productivity. This research finds that the total factor productivity of grain processing enterprises increased by 1.18% annually during the sample period, and that of the central region rose more quickly than the other areas of China. Technical progress contributes more to enterprises’ total factor productivity, but technical efficiency may become a key factor in determining it. The difference in the growth rate of the grain processing enterprises’ total factor productivity among different ones in the eastern and western regions is gradually narrowing, while that of the central region is gradually expanding; there is an obvious technological catch-up effect between and within the regions, especially in the central area of China.

## 1. Introduction

Grain is not only a crucial material basis for human survival, but also an important guarantee for ensuring national security in China. However, achieving grain security is not only dependent on the stability of grain production and stocks, but also closely related to its processing capacity. Since 2004, China’s grain output has grown every year. The grain output was 633 million tons in 2021, an increase of 34.97% compared to 2004. The grain processing industry, as a key link in the entire grain industrial chain, can turn production into supply and achieve a balance between production and sales, which plays an increasingly important role in promoting farmers’ employment and income, as well as agricultural value added and efficiency, and in addition to rural prosperity and development (Zong, 2014) [1]. The grain processing enterprises connect the market and the farmers to achieve an organic combination of production and consumption from the perspective of the industrial chain’s distribution; its development status is directly related to the expansion of the grain processing industry. However, China’s grain processing enterprises are still in the initial stage of growth, and the progress of deep processing in agricultural products is poor and insufficient. There are some issues, such as the small-scale, low-level repeated production and the short industrial chain of agricultural product processing, as well as the “double low” product technology content and added value, etc. The key to improving grain enterprises’ processing capacity is to advance the growth of grain enterprises’ total factor productivity (hereinafter referred to as “TFP”). Improving productivity is the core of enterprise, which is far more important than increasing corporate profits and protecting investors, that is to say, raising grain enterprises’ TFP is the key to boosting grain processing capacity, stabilizing the development of the grain industry, and consequently ensuring grain security (Wu et al., 2017) [2].

China’s grain production is regionalized due to a range of different policies and resource endowment conditions (Gao and Song, 2014; Wu, 2000) [3,4]. Research into grain enterprises’ TFP must consider the heterogeneity characteristics of the regions where the enterprises are located. Compared with other enterprises, the regional characteristics of grain enterprises are mainly related to the market policy environment in which they are situated. For example, the implementation of the food security governor responsibility system further exacerbates the differences in the production efficiency of grain enterprises in different regions (Zhao et al., 2018) [5]. Therefore, how to fully explore and improve the TFP of regional grain enterprises under different market policies and resource endowments is a problem worthy of further study. In other words, how does the TFP of Chinese grain enterprises change and are there regional differences in TFP? How can the existence of this difference be explained? Answering these questions can better improve grain enterprises’ TFP, which is not only related to the survival and advancement of such businesses, but also to the value creation and sustainable development of the entire grain processing sector. Thus, it is of great significance to promote the stable growth of the grain industry.

Existing studies have mainly focused on grain production and rarely considered grain processing. First, with regard to grain productivity, Thirtle et al. (1993) [6] showed that TFP grows at an average rate of 1,3 per cent per annum in South African agriculture for the period from 1947–1991. Kalirajan et al. (1996) [7] found that the TFP growth in Irish agriculture is just over 1% for the period from 1984–2000, but there are significant differences between systems, with sheep systems showing the highest productivity growth, followed by dairy and tillage. Millan et al. (1998) [8], Nghiem et al. (2002) [9], and Rada and Buccola (2012) [10] calculated the total factor productivity of agriculture in Spain, Vietnam, and Brazil, respectively, and found that the average annual growth rate of agricultural TFP was between 2.6% and 3.5%. Some scholars focus on the estimation and comparison of various agricultural sectors’ TFP (Latruffe et al., 2012, 2005; Hadley, 2006) [11,12,13], such as Moldova farms, WA grain farms, Ukrainian agricultural farms, Pakistan’s main food crops, and American agriculture (Plastina and Lence2018; Ali et al., 2016; Balmann et al., 2013; Che et al., 2012; Lerman and Sutton, 2008) [14,15,16,17,18]. Moreover, the increase in total factor productivity caused by technical progress is an important reason for the increase in agricultural output. Eric et al. (2020) [19] found that the TFP growth of Wisconsin dairy producers is estimated at 2.16 per cent, driven primarily by technical progress (1.91 per cent per annum). China’s researchers have examined the estimation and comparison of grain productivity among different regions. Data envelopment analysis (DEA) may be the main method and the following main conclusions are drawn: First, due to the specific sample period selection, variable selection and regional differences could be differences in the trend of grain TFP changes. From a national perspective, Wang and Yang (2021) [20] used the input-output data of grain production in 27 provinces from 1991 to 2016 and found that, although the green total factor productivity of grain in most provinces in the country was in a state of inefficiency, it still shows a trend of gradual growth. Zheng and Cheng (2021) [21] found that the TFP of China’s grain planting industry showed an accelerated growth pattern from 1980 to 2018, with an average annual growth rate of 1.42%. On the contrary, Gao et al. (2021) [22] empirically analysed the temporal and spatial evolution mechanism and technical transmission path of China’s grain total factor productivity from 1999 to 2018, and found that the current grain total factor productivity has a downward trend. From a regional perspective, Zhang and Bao (2016) [23] found that the total factor productivity of China’s main grain producing areas increased by 1.3% annually, and Li and Zhang (2017) [24] found that the total factor productivity of grain in the Yangtze River Economic Belt showed an overall upward trend from 2001 to 2015. From the perspective of provinces, Min (2012) [25] found that the TFP of the overall grain and main varieties in Hubei showed a certain increase from 2004 to 2010. However, Lu et al. (2018) [26] found that, from 2006 to 2016, the total factor productivity of grain in Guangxi decreased by an average of 1.5% per year. Second, technological progress is the main factor affecting the growth of grain TFP. Min and Li (2012) [27] and Gao and Song (2015) [28] concluded that the growth of grain total factor productivity mainly depends on technological progress based on the national grain data from 1978–2010 and 1978–2013, respectively. The same is true based on data from major grain producing areas, planting industries, and a single province (Zheng and Cheng, 2021; Zhao and Yu, 2019; Min and Li, 2012) [21,29,30]. Of course, the lack of motivation for technical progress is also the main reason for the decline in grain TFP (Gao et al., 2021; Lu et al., 2018; Min, 2012) [22,25,26]. Third, there are regional differences in grain TFP. Yin et al. (2016) [31] found that there were regional differences in China’s grain total factor productivity growth from 1978 to 2012, with obvious spatial disequilibrium (Fan, 2016) [32]. Furthermore, Fan (2017) [33] used Kernel density estimation to show that the overall gap in China’s grain total factor productivity was expanding. The difference in grain productivity is also related to the proportion of grain output, whether it is the main grain producing area, and the variety of crops. For example, in the provinces with a high proportion of grain production, grain productivity has shown a state of continuous improvement, while the opposite is true in provinces with a medium and low proportion of grain production (Wang and Lu, 2014) [34]; the total factor productivity performance of the main grain producing areas is generally better than that of the main sales areas and the production and sales balance areas (Wang and Yang, 2021; Lin-Jing et al., 2014) [20,35]. The total factor productivity of rice is higher than that of wheat and maize (Jiang et al., 2016) [36]. Some authors have also measured and compared the grain TFP in China and other countries, and observed that in the countries along the “Belt and Road” it increased from 1995 to 2016, with TP being the main driving force, although there are significant differences in grain TFP among countries, with the fastest rise occurring in Central Asia (Huang and Wei, 2022) [37].

In the grain processing industry, most studies have employed only qualitative research, and there are few empirical examples. Wu et al. (2015) [38] concentrated on the processing enterprise link in the grain circulation process and analysed the welfare changes and output selection of grain processing enterprises under the upstream and downstream differential pricing mechanism. Wu et al. (2017) [2] analysed the technical efficiency and influencing factors of listed companies in China’s grain and oil processing industry based on the three-stage DEA model. The empirical research level has also mainly focused on the rice processing industry. Wu and Xu (2017) [39], based on the industrial enterprise database from 1998 to 2007, used the rice processing industry as an example and found that government subsidies have a negative (positive) impact on capital allocation when the degree of rice price distortion is weak (strong). Specifically in terms of grain processing enterprises, Deng and Xiao (2007) [40] argued that the key to realizing their industrialization is to improve their operation efficiency, and different operation models have various effects on the production efficiency (Lv et al., 2015) [41]. Wang and Wang (2015) [42] applied the DEA model to calculate that the overall efficiency of leading grain enterprises from 2007 to 2011 was generally low. Wang and Wang (2017) [43] utilized the stochastic frontier analysis (SFA) method to calculate that the technical efficiency of leading agricultural enterprises is greater, that it is higher in the central region than in the eastern and western ones, and that in the grain category, it is more advanced than in animal husbandry and other leading enterprises. Zhao et al. (2018) [5] empirically analysed the impact of marketization and government intervention on the TFP of Chinese grain-related enterprises.

The existing literature provides a reference for further analysing the TFP of Chinese grain enterprises for this paper, but there are also some shortcomings. First, most of the existing efficiency analyses in the grain field focus on the production field. There are few studies discussing the efficiency of grain processing enterprises (Han et al., 2014) [44] and the only examples are based on data that relates to, for instance, only a single variety or listed company. Second, most evaluations analyse the influence of internal and external factors on the productivity of grain processing enterprises, and there are also investigations that analyse the TFP of the grain region itself, but there are few that consider whether there are regional differences in the grain enterprises’ TFP sources and whether it converges with time or not. The existing research on the convergence test is mainly aimed at the efficiency of grain production (Yao and Wang, 2019) [45]. Third, most studies do not take into account the actual differences in market environment, technology level, resource endowment, etc., between different regions, which may lead to deviations between the research results and the actual situation.

In view of these deficiencies, based on the data of grain production and processing enterprises from 2014 to 2017, firstly, the meta-frontier Malmquist productivity index in the non-parametric meta-stochastic frontier analysis method was used to measure and decompose grain enterprises’ TFP and examine whether there are regional differences. Secondly, the Dagum Gini coefficient and its decomposition method were used to investigate the regional disparities and their spatial sources of TFP changes in grain enterprises, and explain the existence of regional differences. Finally, we investigated the convergence of the TFP of grain enterprises in the whole country and in various regions with the help of the classical regression model, which tests the variation trend of regional differences over time. The possible contributions of this paper are mainly as follows: First, we used the data of national agricultural industrialization-leading enterprises and took grain processing enterprises as the research object, which not only reflect that food security, including grain security, is very real in China and all over the world, but also reflect that the main task at this stage is to improve the productivity at all stages of the food supply chain, adding new literature to research in this field. Second, we confirm whether the TFP of grain enterprises is in a state of growth, stagnation, or decline, and whether there is a spatial equilibrium in the TFP of grain enterprises, which is directly related to the country’s judgment on the grain processing capacity and the development status of the grain industry. Comparisons, interpretations, and evaluations were carried out at the regional and national levels to have a more comprehensive and systematic understanding of the total factor productivity of Chinese grain enterprises and to better promote the development of the grain industry. Third, we propose that the simplest form of increasing efficiency is exploiting efficiency reserves, and we clearly introduce a method and application that is suitable for helping to exploit productivity reserves.

The structure of this paper is as follows: first, we describe the research question; second, we introduce the methods and data; third, we present the analysis of the regional differences in the TFP of grain processing enterprises; and fourth, we show the judgement of the convergence of the TFP.

## 2. Methods and Data

### 2.1. Methods

TFP is a comprehensive indicator for measuring production efficiency. The traditional DEA method assumes that different regions have common production frontiers and establishes production frontiers to analyse and compare the TE differences of these decision-making units (DMUs). In fact, differences in the market environment, technology level, and resource endowment between regions often affect the speed of technology diffusion; therefore, the technology set available in different regions would inevitably be different, that is, the production frontiers between different regions are not the same. If the productivity is analysed with reference to the respective production technology sets of the sample regions, the analysis results of the sample regions cannot be compared due to the various production frontiers (Li et al., 2013; Wang and Xie, 2012; Chen and Song, 2008) [46,47,48]. The meta-frontier Malmquist index method based on the common frontier analysis framework can effectively solve the above problems.

Hayami and Ruttan (1970) [49] first proposed the conceptual analysis framework of a meta-production function to compare the production efficiency differences of producers under different technical conditions. Ruttan et al. (1978) [50] further regarded the meta-production function as the envelope of the production points of each group, and since then this method has mostly been used to measure agricultural production efficiency (Lau and Yotopoulos, 1989; Mundlak and Hellinghausen, 1982) [51,52]. Since the above meta-production function adopts a fixed boundary form, Sharma and Leung (2000) [53] transformed it into a stochastic meta-frontier production function by assuming that there was a gap between the actual production points of different producers and the production frontier, and that at the same time, the TE value can be estimated, but this TE value cannot be identified by comparison. Therefore, Battese and Rao (2002) [54] overcame this problem by constructing a stochastic common frontier model, but O’Donnell et al. (2008) [55] found that this model did not envelop all the production frontier points. Subsequently, Battese et al. (2004) [56] defined the meta-frontier model as a deterministic boundary that encompasses all group fronts, and proposed the TGR to compare and analyse the differences in production efficiency between regions. Since then, many scholars have extended and applied this method to compare and analyse the growth of TFP in different countries and industries, such as the meta-frontier Malmquist index and the meta-frontier Malmquist–Luenberger index (Wang and Zhu, 2011; Rambaldi et al., 2007; Rao, 2006) [57,58,59].

#### 2.1.1. Malmquist Index

Before introducing the specific research methods of this work, the commonly used productivity index, the Malmquist index (*MI*), is described. *MI* was first introduced by Caves, Christensen, and Diewert in 1982 [60]. Its core idea is to use the observations of each decision-making unit to construct an effective production frontier, which represents the efficient output that can be achieved by various input combinations under certain technical conditions, and then use the distance between each decision-making unit and the production frontier to calculate the production efficiency value (Mcmullen and Okuyama, 2000) [61]. This is also the main advantage of Malmquist approach: that it does not require the assumption of efficient production, but instead identifies the ‘best-practice’ units in countries, regions or farms in every period and measures the distance from that. Let x and y be the input and output vectors, respectively, and the production technology set can be expressed as $T=\left\{\left(x,y\right):x\geq 0;y\geq 0\right\}$, x can produce y. The corresponding output set is $P\left(x\right)=\left\{y:\left(x,y\right)\right\}\in T\}$ and the slacks-based measure (SBM), directional distance function (DDF) based on the group frontier can be expressed as $\vec{D}$ = $\left(x,y;g\right)=sup\left\{\beta :\left(y+\beta y\right)\right\}\in P\left(x\right)\}$. The $MI_{t}$ measuring productivity change, from t to t+1, is defined with respect to a reference period technology; therefore, the *MI* with respect to technology in any t is:

(1) $$MI_{t}=\frac{\vec{D_{t}}\left(x_{k}^{t+1},y_{k}^{t+1};y_{k}^{t+1}\right)}{\vec{D_{t}}\left(x_{k}^{t},y_{k}^{t};y_{k}^{t}\right)}$$

*MI* index of period t+1:

(2) $$MI_{t+1}=\frac{\vec{D_{t+1}}\left(x_{k}^{t+1},y_{k}^{t+1};y_{k}^{t+1}\right)}{\vec{D_{t+1}}\left(x_{k}^{t},y_{k}^{t};y_{k}^{t}\right)}$$

It is difficult to choose between t and t+1 for the reference or benchmark period, so *MI* is generally defined as the geometric mean of (1) and (2) (Chambers et al., 1994) [62]:

(3) $$MI_{t}^{t+1}={\left[\frac{\vec{D_{t}}\left(x_{k}^{t+1},y_{k}^{t+1};y_{k}^{t+1}\right)}{\vec{D_{t}}\left(x_{k}^{t},y_{k}^{t};y_{k}^{t}\right)}\times \frac{\vec{D_{t+1}}\left(x_{k}^{t+1},y_{k}^{t+1};y_{k}^{t+1}\right)}{\vec{D_{t+1}}\left(x_{k}^{t},y_{k}^{t};y_{k}^{t}\right)}\right]}^{\frac{1}{2}}$$

This can be further decomposed into technical efficiency change ($TEC_{t}^{t+1}$) and technical progress change ($TC_{t}^{t+1}$) as follows:

(4) $$MI_{t}^{t+1}=\underset{TEC_{t}^{t+1}}{\underset{︸}{\frac{\vec{D_{t+1}}\left(x_{k}^{t+1},y_{k}^{t+1};y_{k}^{t+1}\right)}{\vec{D_{t}}\left(x_{k}^{t},y_{k}^{t};y_{k}^{t}\right)}}}\underset{TC_{t}^{t+1}}{\underset{︸}{{\left[\frac{\vec{D_{t}}\left(x_{k}^{t+1},y_{k}^{t+1};y_{k}^{t+1}\right)}{\vec{D_{t+1}}\left(x_{k}^{t+1},y_{k}^{t+1};y_{k}^{t+1}\right)}\times \frac{\vec{D_{t}}\left(x_{k}^{t},y_{k}^{t};y_{k}^{t}\right)}{\vec{D_{t+1}}\left(x_{k}^{t},y_{k}^{t};y_{k}^{t}\right)}\right]}^{\frac{1}{2}}}}$$

Thus, it can be seen that the *MI* lends itself to a decomposition into *TE* change and *TP* change (Grosskopf, 2003) [63]; where, *TE* and *TP* are the abbreviations for technical efficiency and technical progress, respectively.

#### 2.1.2. Malmquist Index under Group Frontier and Meta-Frontier

Assuming that the sample can be divided into different groups according to a certain standard, the feasible input-output combinations of all decision-making subjects under each group belong to the same production technology set, and there are g group technology sets in the sample (*g* = 1, 2, *…*, *G*). The production technology set of the *G*-th group frontier can be expressed as $T^{G}=\left\{x,y:x\geq 0;y\geq 0\right\}$, x can produce y, its corresponding output set is $P^{G}$(*x*), and the SBM DDF based on the group frontier can be expressed as $\vec{D_{G}}$ = $x,y;g=\sup\left\{\beta :y+\beta y\in P^{G}\left(x\right)\right\}$. Therefore, the group frontier Malmquist index (hereafter *GMI*) can be defined with reference to Formula (4) as follows:

(5) $$GMI_{t}^{t+1}=\frac{\vec{D_{G}^{t+1}}\left(x_{k}^{t+1},y_{k}^{t+1};y_{k}^{t+1}\right)}{\vec{D_{G}^{t}}\left(x_{k}^{t},y_{k}^{t};y_{k}^{t}\right)}\times {\left[\frac{\vec{D_{G}^{t}}\left(x_{k}^{t+1},y_{k}^{t+1};y_{k}^{t+1}\right)}{\vec{D_{G}^{t+1}}\left(x_{k}^{t+1},y_{k}^{t+1};y_{k}^{t+1}\right)}\times \frac{\vec{D_{G}^{t}}\left(x_{k}^{t},y_{k}^{t};y_{k}^{t}\right)}{\vec{D_{G}^{t+1}}\left(x_{k}^{t},y_{k}^{t};y_{k}^{t}\right)}\right]}^{\frac{1}{2}}$$

where $\frac{\vec{D_{G}^{t+1}}\left(x_{k}^{t+1},y_{k}^{t+1};y_{k}^{t+1}\right)}{\vec{D_{G}^{t}}\left(x_{k}^{t},y_{k}^{t};y_{k}^{t}\right)}$ is the *TE* index, namely group frontier efficiency change t to t+1($GEC_{t}^{t+1}$) and ${\left[\frac{\vec{D_{G}^{t}}\left(x_{k}^{t+1},y_{k}^{t+1};y_{k}^{t+1}\right)}{\vec{D_{G}^{t+1}}\left(x_{k}^{t+1},y_{k}^{t+1};y_{k}^{t+1}\right)}\times \frac{\vec{D_{G}^{t}}\left(x_{k}^{t},y_{k}^{t};y_{k}^{t}\right)}{\vec{D_{G}^{t+1}}\left(x_{k}^{t},y_{k}^{t};y_{k}^{t}\right)}\right]}^{\frac{1}{2}}$ is the *TP* index, namely group frontier technology change from t to t+1($GTC_{t}^{t+1}$). That is to say, the TFP index of the decision-making subjects under the *G*-th group frontier in the t to t+1 can be decomposed into a *TE* index and a *TP* index, as follows:

(6) $$GMI_{t}^{t+1}=GEC_{t}^{t+1}\times GTC_{t}^{t+1}$$

Since the meta-frontier is regarded as a frontier production function formed by enveloping all group frontiers, the meta-frontier technology set can be expressed as $T^{M}=\left\{T^{1}\cup T^{2}\ldots \ldots \cup T^{G}\right\}$ and satisfies the closed, bounded, and convex assumptions, and its corresponding output set is P^M (x), and the DDF of the SBM based on the common front can be expressed as $\vec{D_{M}}$ = $x,y;g=\sup\left\{\beta :y+\beta y\in P^{M}\left(x\right)\right\}$. Therefore, the meta-frontier Malmquist index (*MMI*) can also be defined with reference to Formula (4) as follows:

(7) $$\;MMI_{t}^{t+1}=\frac{\vec{D_{M}^{t+1}}\left(x_{k}^{t+1},y_{k}^{t+1};y_{k}^{t+1}\right)}{\vec{D_{M}^{t}}\left(x_{k}^{t},y_{k}^{t};y_{k}^{t}\right)}\times {\left[\frac{\vec{D_{M}^{t}}\left(x_{k}^{t+1},y_{k}^{t+1};y_{k}^{t+1}\right)}{\vec{D_{M}^{t+1}}\left(x_{k}^{t+1},y_{k}^{t+1};y_{k}^{t+1}\right)}\times \frac{\vec{D_{M}^{t}}\left(x_{k}^{t},y_{k}^{t};y_{k}^{t}\right)}{\vec{D_{M}^{t+1}}\left(x_{k}^{t},y_{k}^{t};y_{k}^{t}\right)}\right]}^{\frac{1}{2}}\;=MEC_{t}^{t+1}\times MTC_{t}^{t+1}$$

where $MMI_{t}^{t+1}$ (meta-frontier Malmquist change), $MEC_{t}^{t+1}$ (meta-frontier efficiency change), and $MTC_{t}^{t+1}$ (meta-frontier technology change) represent the meta-frontier TFP index, meta-frontier technical efficiency index, and meta-frontier technical progress index of the DMU to be evaluated t to t+1, respectively. Drawing on the research of Rambaldi et al. (2007) [57], $MEC_{t}^{t+1}$ and $MTC_{t}^{t+1}$ are further decomposed:

(8) $$MEC_{t}^{t+1}=\frac{\vec{D_{G}^{t+1}}\left(x_{k}^{t+1},y_{k}^{t+1};y_{k}^{t+1}\right)}{\vec{D_{G}^{t}}\left(x_{k}^{t},y_{k}^{t};y_{k}^{t}\right)}\times \frac{TGR^{t+1}\left(x_{t+1},y_{t+1}\right)}{TGR^{t}\left(x_{t},y_{t}\right)}={\mathrm{GEC}}_{t}^{t+1}\times {\mathrm{PTCU}}_{t}^{t+1}$$

(9) $${\mathrm{MTC}}_{t}^{t+1}={\left[\frac{\vec{D_{G}^{t}}\left(x_{k}^{t+1},y_{k}^{t+1};y_{k}^{t+1}\right)}{\vec{D_{G}^{t+1}}\left(x_{k}^{t+1},y_{k}^{t+1};y_{k}^{t+1}\right)}\times \frac{\vec{D_{G}^{t}}\left(x_{k}^{t},y_{k}^{t};y_{k}^{t}\right)}{\vec{D_{G}^{t+1}}\left(x_{k}^{t},y_{k}^{t};y_{k}^{t}\right)}\times \frac{{\mathrm{TGR}}^{t}\left(x_{t},y_{t}\right)}{{\mathrm{TGR}}^{t+1}\left(x_{t+1},y_{t+1}\right)}\times \frac{{\mathrm{TGR}}^{t}\left(x_{t+1},y_{t+1}\right)}{{\mathrm{TGR}}^{t+1}\left(x_{t},y_{t}\right)}\right]}^{\frac{1}{2}}\;={\mathrm{GTC}}_{t}^{t+1}\times {\mathrm{PTRC}}_{t}^{t+1}$$

where ${\mathrm{PTCU}}_{t}^{t+1}$ is pure technology catch-up, reflecting the change in the DMU TGR and $PTRC_{t}^{t+1}$ is the relative change in potential technology, representing the relative movement speed of the meta-production frontier.

Therefore, the *MMI* index under the meta-frontier is further decomposed as follows:

(10) $$\begin{array}{c} MMI_{t}^{t+1}=GMI_{t}^{t+1}\times \frac{MMI_{t}^{t+1}}{GMI_{t}^{t+1}}=GEC_{t}^{t+1}\times GTC_{t}^{t+1}\times \frac{MEC_{t}^{t+1}}{GEC_{t}^{t+1}}\times \frac{MTC_{t}^{t+1}}{GTC_{t}^{t+1}}\\ =GEC_{t}^{t+1}\times GTC_{t}^{t+1}\times PTCU_{t}^{t+1}\times PTRC_{t}^{t+1} \end{array}$$

It can be seen that the *TE* may be the key part, and we used the super-efficiency SBM model to measure the *TE*. In the radial data envelopment analysis model (DEA) model, the measurement of the degree of inefficiency only includes the proportional reduction (increase) of all inputs (outputs), and the measurement of the efficiency value does not reflect the slack improvement. Tone (2011) [64] proposed the super-efficiency SBM model in 2001, which is a non-radial and non-angular relaxation-based efficiency evaluation model; the obtained efficiency value decreases with the increase of the relaxation input and output, and the efficiency evaluation standard is based on the reference set and depends only on the decision unit to be considered, between 0 and 1, with the formula as follows:

(11) $$\begin{array}{c} Min\rho_{SE}=\frac{1+\frac{1}{m}\sum_{i=1}^{m}\frac{s_{i}^{-}}{x_{ik}}}{1-\frac{1}{s}\sum_{r=1}^{s}\frac{s_{r}^{+}}{y_{rk}}} \end{array}\;s.t.\;\sum_{j=1,j\neq k}^{n}x_{ij}\lambda_{j}-s^{-}\leq x_{ik},\;\sum_{j=1,j\neq k}^{n}y_{ij}\lambda_{j}+s^{+}\geq y_{rk},\;\lambda ,s^{-};s^{+}\geq 0;I=1,2,\ldots ,m;r=1,2,\ldots ,s;j=1,2,\ldots n\;(j\neq k)$$

where $\rho_{\mathrm{SE}}$ is the efficiency value of super-efficiency SBM, X and Y represent input and output variables, respectively—where the number of input indicators is m and the number of output indicators is s—$s^{-},s^{+}$ are the input and output slack variables, respectively, and is the weight vector. When $\rho_{\mathrm{SE}}\geq$1 and $s^{-}=s^{+}$ = 0, the DMU is relatively effective for DEA; when $\rho_{\mathrm{SE}}\geq$ 1, but $s^{-},s^{+}$ not all 0, the DMU is weakly effective for DEA. When $\rho_{\mathrm{SE}}$ is less than 1, it means that the DMU is relatively ineffective for DEA, indicating that there is redundancy and the input and output need to be improved to bring the DMU to an effective level.

### 2.2. Indicator Selection

We selected the enterprise sales income as the output indicator, which can reflect the enterprise’s production operation and size. In terms of input indicators, capital investment was expressed by the total assets of the enterprise in the current year. Labour input was represented by the number of employees in the current year. In the production process of agricultural enterprises, enterprises mainly employ two types of workers, perennial and seasonal, and we mainly considered the perennial category. The number of intermediate inputs was illustrated by the enterprise’s raw material input. The statistical description of the variables is shown in Table 1.

### 2.3. Data

The sample used in this paper is composed of the grain enterprises of the national leading agricultural industrialization enterprises, mainly in production and processing types, including the data of 245 leading grain agricultural enterprises (hereinafter referred to as “grain enterprises”) from 2014 to 2017. A total of 980 sample annual data points were finally obtained, where the number of grain enterprises in the eastern, central, and western regions of China was 75, 102 and 68, respectively. Due to the lack of data on enterprises in Tibet of China, we only studied the grain enterprises in the remaining 30 provinces, and in the process of evaluating the leading national agricultural industrialization enterprises, Hainan was included in the western region with reference to the standards of the national leading enterprises in the western region. Therefore, the eastern region includes Beijing (BJ), Tianjin (TJ), Hebei (HE), Liaoning (LN), Shanghai (SH), Jiangsu (JS), Zhejiang (ZJ), Fujian (FJ), Shandong (SD), and Guangdong (GD); the central region includes Shanxi (SX), Jilin (JL), Heilongjiang (HL), Anhui (AH), Jiangxi (JX), Henan (HA), Hubei (HB), and Hunan (HN); and the western region includes Chongqing (CQ), Sichuan (SC), Guizhou (GZ), Yunnan (YN), Shaanxi (SN), Gansu (GS), Qinghai (QH), Ningxia (NX), Xinjiang (XJ), Guangxi (GX), Inner Mongolia (IM), and Hainan (HI). This research is based on the input-oriented Malmquist index method to measure the TFP of grain enterprises in the eastern, central, and western regions of China, and the software used is MaxDEA6.7.

## 3. Calculation of Grain Enterprises’ TFP in China

### 3.1. Trends and Regional Disparities of Grain Enterprises’ TFP Index

Based on the meta-frontier Malmquist productivity index, the changing trend of the meta-frontier TFP of grain enterprises across the country and in the three regions from 2014 to 2017 is calculated, as shown in Table 2. At the national level, the TFP of China’s grain enterprises showed a trend of growth. The average annual growth rate of TFP was 1.18%, and the cumulative growth rate was 3.54% from 2014 to 2017.The average values of the TE index and the TP index of grain enterprises were 0.9916 and 1.0218, respectively, indicating a pattern of the deterioration of TE and the enhancement of TP in China’s grain enterprises. Since 2014, the TFP improvement of Chinese grain enterprises has been mainly driven by TP, which has surpassed the decline of TE and played a central role in TFP development. The TFP growth of grain enterprises was mainly the “outward shift” of the production frontier rather than the “catch-up” to the production frontier. The contribution of TE and TP to the TFP increase in grain enterprises was not stable; the contribution of TE fluctuated and decreased from 2014 to 2017, while TP showed a fluctuating upward trend. The contribution of TE was greater than that of TP before 2015 whereas, after 2015, the contribution of TP has been even more significant. This conclusion is consistent with the one drawn by Gao (2015) [65] using agricultural industry data. Rahman and Salim (2013) [66] found that TFP grew at an average rate of 0.57% p.a. from 1948–2008, and it was largely powered by technical progress estimated at 0.74% p.a., based on data from 17 regions of Bangladesh. Gautam and Yu (2015) [67] found that the agriculture sector performed impressively with annual TFP growth beyond 2 percent in China and between 1 and 2 percent in India since the 1980s, the TFP growth is mainly propelled by technological progress. However, Candemir and Deliktas (2009) [68] found that the total factor productivity decreased 1.2 percent due to a 2.7 percent technical regress over the 1999–2003 period based on data from Turkish State Agricultural Enterprises, and Lissitsa and Odening (2015) [69] based on the data of Ukrainian agricultural enterprises, concluded that from 1990–1999, TFP declined by 6% annually, dropping a total 42%. The main reason for the observed TFP decline is a decrease in technical efficiency.

In terms of sub-regions, the TFP index values of grain enterprises in the eastern, central, and western regions of China from 2014 to 2017 were all greater than 1, indicating that the development level of grain enterprises has improved, which is coupled with the overall increase in their TFP. The TFP values of those in the central, western and eastern regions each consequently decreased, with annual growth rates of 1.41%, 1.15%, and 0.90%, respectively. There are also conspicuous variances in the same cumulative growth rates of TFP, which are 2.70%, 4.22%, and 3.44% in the eastern, central, and western regions, respectively. The meta-frontier TE of grain enterprises in the eastern, central, and western regions all showed a downward trend, but the decline rate was essentially 0.85%; the TP all showed an increasing trend, with an increase rate of about 2.16%, indicating that TP is the core factor leading to the improvement of the TFP of grain enterprises in various regions. It is worth noting that from 2016 to 2017, grain enterprises in the eastern, central, and western regions all showed a trend of deterioration of TE and technological regression, where the deterioration of TE accounted for 79.25%, 84.62%, and 70.59% of the decline in the TFP of grain enterprises, respectively, and the proportion of technical regression was 20.75%, 7.69%, and 29.41%, respectively. It can be seen that the proportion of TE deterioration is much greater than that of technological regression. Therefore, improving the level of TE is an important method of promoting the growth of grain TFP. Pratt et al. (2009) [70] compared TFP growth in China and India and found that efficiency improvement played a dominant role in promoting TFP growth in China. Alem (2017) [71] indicated that TFP increased by 0.03 and 0.01 percent per annum for the eastern and central regions, respectively; and technical change has had the main source of productivity change based on Norwegian grain production sector data.

**Figure 1:**
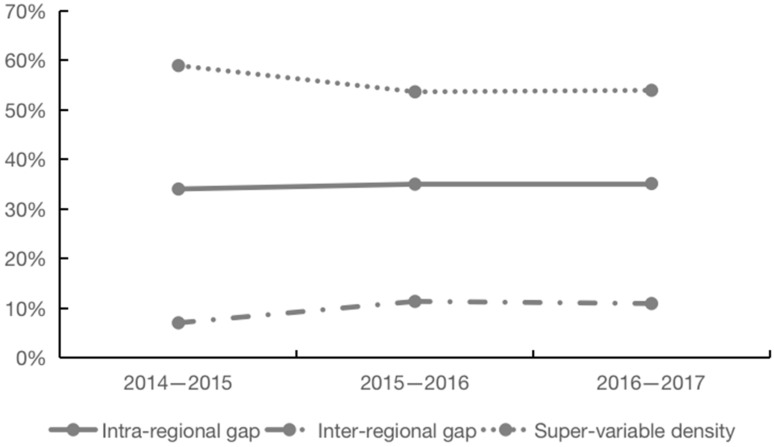
Spatial source of regional gap in grain enterprises’ TFP from 2014–2017.

Why are there differences in the TFP of grain enterprises in different regions? Based on MATLAB software, we adopted the Dagum Gini coefficient and its decomposition method to investigate the regional gap and spatial source of the TFP changes of grain enterprises. First, the regional disparity in the growth of Chinese grain enterprises’ TFP showed a trend of rising and then falling in an overall slight decline, with a drop of only 0.04%. The regional gap in TFP growth before 2016 widened rapidly, rising from 0.0083 in 2014 to 0.0172 in 2016, with an average annual growth rate of 0.45%; during this period, the production levels of grain enterprises in various regions were uneven, resulting in the regional gap in TFP continuing to expand. Since then, the regional gap of the TFP of grain enterprises has gradually narrowed and the regional gap of TFP in 2017 was 0.0079. Second, the super-variable density of the TFP growth of grain enterprises has been the main source of the overall spatial gap, with an average annual contribution rate of 55.53%, followed by the intra-regional (34.71%) and inter-regional ones (9.76%), as shown in Figure 1. The contribution rate of the intra-regional disparity in China’s grain enterprises’ TFP is gradually increasing, which also implies that the non-equilibrium growth of grain enterprises’ TFP is mainly due to the differences within the three regions. Improving the growth potential of TFP in the eastern, central, and western regions and narrowing the intra-regional gap of TFP growth are the keys to promoting the coordinated growth of the TFP of grain enterprises.

According to the distribution differences of the cumulative TFP of grain enterprises in the remaining 30 provinces in 2017. It can be known that the top six provinces with the fastest growth in the cumulative TFP of grain enterprises are Jilin, Anhui, Guangxi, Hebei, Shaanxi, and Hunan, with growth rates ranging from 4.53% to 6.42%; the last six provinces with the slowest growth are Shanxi, Liaoning, Shandong, Hubei, Fujian and Guizhou, where the cumulative TFP growth rates is within the range of 0.23% to 2.11%. In addition, Jilin and Hubei, both belonging to the central region, ranked first and 28th, respectively, among the 30 provinces in terms of cumulative TFP, while Hebei and Fujian, both belonging to the eastern region, ranked fourth and 29th, respectively, and Guangxi and Guizhou, both of which belong to the western region, ranked second and 30th, respectively. It can be seen that there is a large difference in TFP growth among provinces in the same region.

### 3.2. Group Decomposition of Grain Enterprises’ TFP Index

According to Formulas (8) and (9), the meta-frontier TFP of grain enterprises can be further decomposed into inter-group indices and indices with specific economic meanings, which can be used to analyse the source differences of the TFP changes of grain enterprises in different regions, and the specific decomposition results are shown in Table 3. The results show the following: First, the GEC, the TE of grain enterprises in China as a whole and in the three major regions, showed a fluctuating downward trend, with an average annual growth rate of −0.97%, −1.32%, −0.69%, and −1.00%, respectively. The central, western, and eastern regions consequently decreased. The drop in TE indicates that there is still more room to promote the TFP growth of grain enterprises by improving TE; this also implies that the advancement of grain enterprises still faces many problems, such as high production costs as well as unreasonable resource allocation, weak business management ability, and high market competition pressure, etc. Due to the lack of corresponding policy support and encouragement, enterprises would be delisted while reducing their production enthusiasm. Second, in terms of the group frontier technology change (GTC), the TP of grain enterprises in China as a whole and in the three major regions has accelerated, with an average annual growth rate of 2.21%, 2.73%, 1.80%, and 2.25%, respectively. This shows that most agricultural enterprises have made breakthroughs in technological innovation as well as research and development, especially in the western region. Third, the average PTCU value of grain enterprises in China as a whole and the three major regions are 1.0020, 1.0045, 1.0002, and 1.0020, respectively, and the PTCU value in most years is also greater than 1, indicating that the meta-frontier technology efficiency index of grain enterprises is greater than the TE index of the group frontier; that is, there is a catch-up effect between the actual production technology of DMU produced by grain enterprises and the optimal DMU on the meta-frontier and the gap between the two is gradually narrowing. Fourth, the average potential technological relative change (PTRC) values of China’s overall and three major regional grain enterprises are 0.9998, 0.9936, 1.0052, and 0.9987, respectively. It can be seen that, except for the central region, the MTC of China as a whole, as well as the eastern and western regions, is smaller than the GTC, that is to say, the group frontier technology progress is greater than the meta-frontier technology progress; and the PTRC value is less than 1 in most years, indicating that the meta-technology frontier of grain enterprises moves relatively fast, which also means that there is great space and potential for the TP of grain enterprises. Furthermore, the differences in GEC between eastren region and central region, central region and western region, eastern region and western region are −0.63%, 0.31%, and −0.32%, respectively, and those in MMI are −0.51%, 0.26%, and −0.25%, respectively. There is a positive correlation between the GEC and MMI, indicating that the main reason for the differences in grain enterprises’ TFP between regions is the variances in TE among the three regions in China. Karafillis and Papanagiotou (2008) [72] concluded that the rate of technical difference is always positive in the formulation of TFP difference based on the data of organic olive enterprises in the Greek region.

### 3.3. Analysis of the TE of Grain Enterprises in China

From the decomposition of the meta-frontier TFP index and the group frontier TFP index of grain enterprises from 2014 to 2017, we can observe the following: First, the deterioration of TE has seriously affected the growth of the TFP of grain enterprises. Second, in the years when both the TE index and the TP index declined, the effect of TE deterioration on the TFP of enterprises was much greater than that of technological regression. Finally, the differences in TE between regions directly lead to the regional variances in the TFP of grain enterprises. Therefore, it is necessary to measure and analyse the TE of grain enterprises in various regions, so as to promote the TFP growth of grain enterprises by improving the TE. We used the SBM super-efficiency model with variable returns to scale (VRS) to estimate the comparative changes in the technical efficiency of the meta-frontier and group frontier of grain enterprises from 2014 to 2017; the results are shown in Table 4.

From the perspective of changes in the meta-frontier technological efficiencyof grain enterprises, there are differences in grain enterprises’ MTE in different regions of China. The average TE of grain enterprises in the central region is higher than that in the eastern and western regions, indicating that the implementation and management of agricultural technology and the input and use efficiency of resource elements in the central region are better than those in the eastern and western ones. In addition, the technical inefficiencies in the eastern, central, and western regions are 13.95%, 13.37%, and 14.27%, respectively, which means that there are different degrees of resource waste or insufficient output in the three regions. It is necessary to reduce production inputs while maintaining existing outputs, or to maximize outputs while maintaining existing inputs. From the perspective of time trends, the average annual decline rates of TE in the eastern, central, and western regions are 0.99%, 0.82%, and 0.84%, respectively. Although the TE of the east is higher than that of the western region, the decline rate of the TE of the east is higher than that of the west, and the TE gap between the eastern region and the western region gradually narrow over time. At the same time, the decline rate of TE in the central region is the lowest, indicating that the absolute gap in TE between the central region and the eastern and western regions become increasingly large. The overall trend of TE in the three regions also clearly shows the differences in the production of grain enterprises in different regions; with the continuous advancement and development of urbanization and agricultural modernization in recent years, the regional function of grain production has become increasingly prominent and the advantages of grain production in the main production areas are conspicuous. In the central region, most provinces belong to the main grain production areas, so the level of TE in the region is also higher.

Table 4 compares the trends of technological efficiency of grain enterprises across the country and the three regions under the group frontier and the meta-frontier. Under the group frontier, it is assumed that the grain enterprises in each region face the production frontier composed of their respective groups, and their efficiency values reflect the input-output level under their respective production technology conditions. The results show that the region with the actual production point closest to its own production frontier is the west, followed by the centre and the east. Because the group frontier leads to a lack of comparability of TE between regions and under the meta-frontier, the grain enterprises in each region are faced with the potential optimal production frontier composed of the three regions, which makes the obtained TE value more comparable. The results show that the central TE is the highest, followed by the eastern and western regions. At the same time, the technology sets of grain enterprises in the eastern and central regions are essentially the same under the two frontiers, so the efficiency value does not change much, and the difference is not obvious. However, the technology sets of grain enterprises in the whole country and in the western region under the two frontiers are quite different; therefore, the TE values of grain enterprises belonging to these two groups are overestimated at the frontier of their respective groups, especially in the western region, which is seriously overestimated.

**Figure 2:**
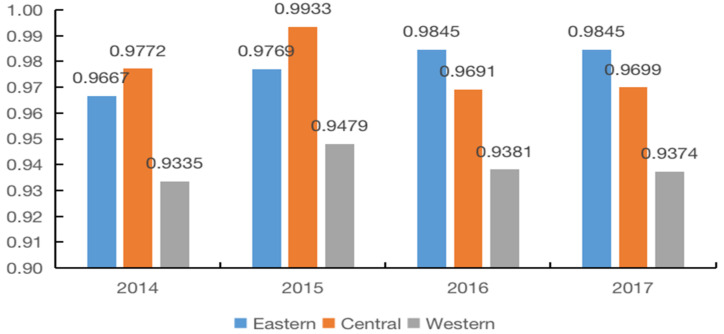
The production TGR of grain enterprises from 2014 to 2017.

The TGR can be obtained from the ratio of the meta-frontier technical efficiency (MTE) to the group frontier technical efficiency (GTE), which is used to reflect the deviation of the actual production technology level from the common frontier. Judging from the overall change trend of TGR (see Figure 2), TGR in the eastern and western regions showed a fluctuating upward trend, indicating that the distance between the production frontier and the meta-frontier in the eastern and western regions is narrowing, and that the gap between the actual production technology and the most advanced production technology is shrinking. The TGR in the central region showed a fluctuating downward trend, with a decline rate of 0.73%, suggesting that the distance between the production frontier in the central region and the meta-frontier has slightly expanded, which means that the gap between the production technology in the central region and the most advanced production technology is widening. The eastern region has the highest annual average TGR at 0.9781, implying that the technical level of the eastern region is higher than that of the central and western ones, and has a significant relationship with the higher economic development level and marketization level of the eastern region. The average TGR in the central region is 0.9774, which means that its production technology level is also high, achieving 97.74% of the optimal technology level. In contrast, the technical level of grain enterprises in the western region is the lowest, and the average TGR is only 0.9392, which is still 6.08% behind the optimal production technology level, indicating that the grain enterprises in the western region have great potential for future production technology improvement. This conclusion is consistent with that obtained by Li et al. (2013) [47], that is, the annual average value of TGR in the eastern region is the highest, followed by the central region, and the value of TGR is the lowest in the western region, which are 0.988, 0.962, and 0.853, respectively.

## 4. Convergence of Grain Enterprises’ TFP in China

There are differences in the grain enterprises’ TFP in different regions of China. During the sample period, the gaps in TE within the region gradually narrow, and those in the technological gap ratios of grain enterprises between the east and the centre, between the east and the west, and between the centre and the west show a trend of first widening and then narrowing. So, with the passage of time, will the TFP of food enterprises converge? Are regional differences increasing or shrinking? That is to say, another question studied in this work was whether the TFP growth of grain enterprises has the convergence phenomenon considered by the neoclassical economic theory.

TFP convergence research has gradually attracted the attention of scholars (Miller and Upadhyay, 2002; Sala-i-Martin, 1996; Islam, 1995) [73,74,75]. σ convergence means that the variance, standard deviation or coefficient of the variation of output or income levels in different regions is shrinking, focusing on the comparison of indicators from a horizontal angle. β convergence means that the growth rate of individuals with lower levels at the beginning of the period is higher than that of those with higher levels at the beginning of the period, reflecting the speed of regional economic development or production efficiency, which is negatively correlated with the initial level, and focuses on the comparison of indicators from a longitudinal perspective, including absolute β convergence and conditional β convergence. Absolute β convergence means that, assuming that each region has the same economic conditions, the development level of grain enterprises in different regions converge to the same steady state with time; conditional β convergence means that, under different economic conditions, the development level of grain enterprises in each region converge to their own steady state. Based on this, we calculate the convergence of grain enterprises’ TFP in the whole country and the eastern, central, and western regions and the spatial correlation of grain enterprises’ TFP in various provinces. The software used is stata15.0.

### 4.1. σ Convergence and β Convergence of Grain Enterprises’ TFP

**Figure 3:**
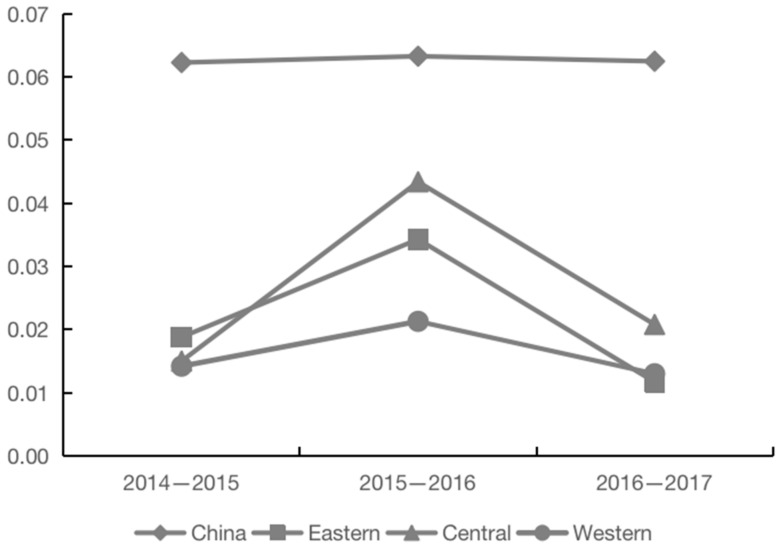
The evolution trend of the coefficient of the variation in grain enterprises’ TFP growth.

In this research, we used the coefficient of variation to examine the convergence of grain enterprises’ TFP, and the results are shown in Figure 3. The growth of grain enterprises’ TFP in the eastern, central, and western regions shows roughly the same characteristics of convergence and divergence, that is, it tends to diverge from 2014 to 2016, and to converge from 2016 to 2017. However, there are differences in the convergence and divergence from 2014 to 2017, among which the eastern and western regions have obvious convergence trends, and the central region has obvious divergent ones. This shows that the difference in the TFP growth rate of grain enterprises between different enterprises in the eastern and western regions is gradually narrowing, while that in the central region is gradually expanding. On the whole, the coefficient variation of grain enterprises’ TFP variation across the country is very small and there are no obvious convergence and dispersion characteristics, indicating that there is an imbalance in the growth of grain enterprises’ TFP in various regions.

The empirical test results of Model 1 further verify the above results. As shown in Table 5, the TFP growth of grain enterprises in the eastern and western regions of China has a significant convergence, while in the central region, there is a significant divergence and the TFP growth of the national grain enterprises also tends to diverge, but not significantly. Although TFP growth in the whole country and the central region does not demonstrate convergence, there may be β convergence. The results of Model 2 show that the TFP growth of grain enterprises in the whole country and the eastern, central, and western regions has a significant absolute β convergence, indicating that there is an obvious technological catch-up effect between and within regions, and the convergence rate of the TFP growth of grain enterprises in the central region is higher than that in the western and eastern ones, indicating that the technology related to grain production has been better diffused in the central one. Since the absolute β convergence test is based on the assumption that each economy has the same economic conditions, the growth rate and growth level of grain enterprises’ TFP converges with the passage of time. The test results of Model 3 further illustrate the regional differences in the TFP growth of grain enterprises. There is a significant conditional β convergence in the TFP growth of grain enterprises in the whole country, as well as the eastern, central, and western regions, and the convergence speed of the TFP growth of grain enterprises in the central, eastern, and western regions consequently decreases, that is, the time required for the growth of grain enterprises’ TFP to reach their respective steady-state levels consequently increases.

To sum up, the TFP of grain enterprises in the eastern, central, and western regions all showed an increasing trend, and the growth of TFP has both absolute β convergence and conditional β convergence, which shows that the TFP gap between the three regions is narrowing and the efficiency level of grain enterprises within the region is also gradually tending to a steady state. The explanation for the convergence phenomenon of the TFP growth of grain enterprises is that, under the current marketization of factor resources, the open market environment allows and encourages the flow of factors among different regions. At the same time, the informatization and marketization of production technology also leads to the convergence of the level of technology acquisition. Therefore, there would be a trend of productivity convergence in different regions (Ma et al., 2015) [76]. Baldoni and Esposti (2020) [77] investigated the scale, scope, and nature of the spatial dependence of agricultural TFP based on Italian FADN farm-level data for the period from 2008–2015 and found that agricultural productivity spill-overs significantly occur though over a limited spatial range.

### 4.2. Spatial Correlation of Grain Enterprises’ TFP

In order to examine the spatial dependence and spatial agglomeration of the TFP growth of grain enterprises, we further obtained Moran’s I value of the TFP growth of grain enterprises in various regions from 2014 to 2017 based on the cumulative TFP index of each province to investigate the spatial correlation of grain enterprises’ TFP. Moran’s I value is distributed between [−1, 1]. A value greater than 0 indicates positive spatial correlation; less than 0 indicates spatial negative correlation; equal to 0 is uncorrelated, that is, randomly distributed in space; and the absolute value of Moran’s I measures the degree of spatial correlation. The larger (smaller) the absolute value, the stronger (weaker) the spatial correlation (Liu and Jia, 2019) [78].

Table 6 shows the calculation results of Moran’s I value of the TFP of grain enterprises from 2014 to 2017. The TFP growth of grain enterprises in China’s various provinces is negatively correlated and the degree of spatial correlation gradually increases with time. The absolute value of Moran’s I gradually increased from 2015 to 2017, which were 0.171, 0.212, and 0.270 over the three years, respectively.This shows that the spatial negative correlation of the TFP growth of grain enterprises increases significantly over time, that is to say, those with the same attribute value are less likely to cluster together and the degree of factor flow and technical exchange between adjacent regions needs to be further deepened.

**Figure 4:**
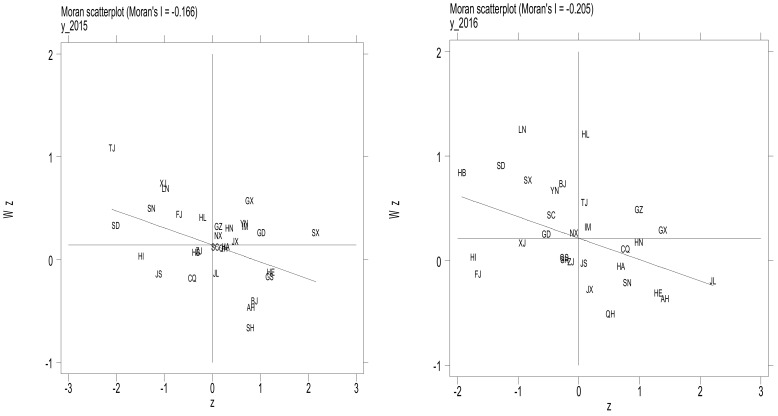

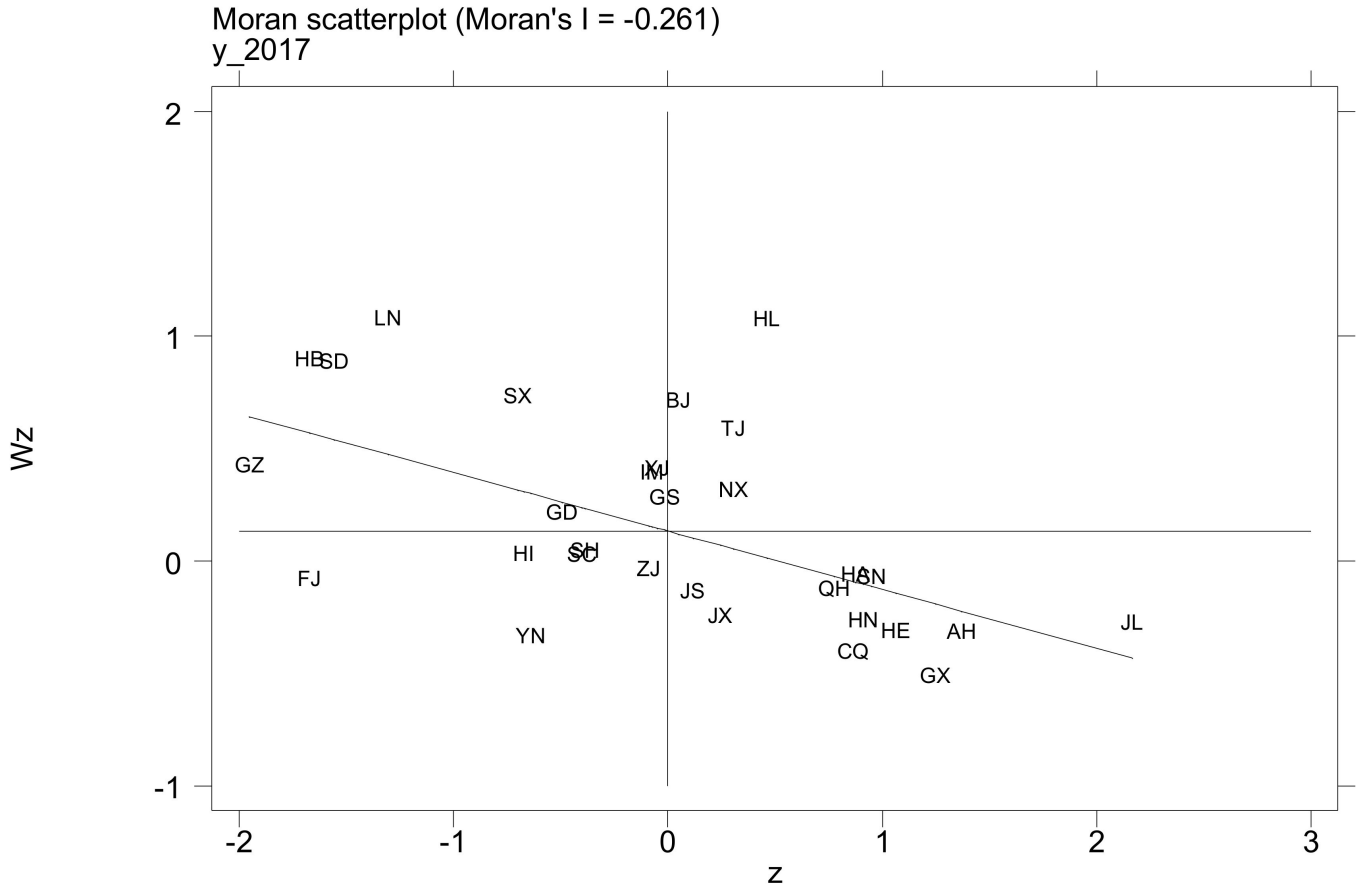
Moran scatter plot of grain enterprises’ TFP.

Figure 4 further shows the Moran scatter plot of the TFP growth of grain enterprises in 30 provinces in China from 2015 to 2017, in order to examine the spatial agglomeration pattern of the TFP growth of grain enterprises. The four quadrants of the Moran scatter plot represent high and high agglomeration (H-H agglomeration), low and high agglomeration (L-H agglomeration), low and low agglomeration (L-L agglomeration), and high and low agglomeration (H-L agglomeration), respectively, in which H-H and L-L clustering represent positive spatial correlation, and L-H and H-L clustering represent negative spatial correlation (Liu et al., 2020) [79]. It can be seen that in 2017, more than 60% of the provinces were located in the second and fourth quadrants, such as GZ, HB, SD, LN, SX, GD, JS, JX, QH, HA, SN, HN, HE, CQ, AH, GX, and JL; other provinces are located in the first and third quadrants. This shows that the spatial agglomeration pattern of the TFP growth of grain enterprises in various provinces in China is mainly L-H and H-L agglomeration: either low values are surrounded by high ones, or high values are surrounded by low ones, demonstrating a negative spatial correlation as a whole.

## 5. Research Conclusions and Policy Implications

We measured and decomposed the TFP of 245 grain enterprises in the eastern, central, and western regions from 2014 to 2017 under the two fronts of the non-parametric meta-frontier and the group frontier; compared and analysed the regional differences in grain enterprises’ TFP; and tested the convergence of eastern, central, and western grain enterprises’ TFP. The following conclusions were drawn: First, the TFP of China’s grain processing enterprises shows a growth trend, and the regional gap is obvious, mainly due to the differences within the region. The average annual growth rate of grain enterprises’ TFP in China and the eastern, central, and western regions was 1.18%, 0.90%, 1.41%, and 1.15%, respectively. The growth rate of grain processing enterprises’ TFP in the central region was significantly higher than that in the eastern and western ones. The regional disparity in the growth of grain enterprises’ TFP showed an evolution trend of first rising and then decreasing and it was mainly caused by hyper-variable density, followed by intra-regional disparity. Second, the TFP growth of Chinese grain enterprises is driven by TP. All Chinese grain enterprises have a development pattern in which TE deteriorates and TP increases, but the contribution of TE is greater than that of TP from 2014–2015, and the proportion of TE deterioration in the decline in the TFP of grain enterprises from 2016–2017 is much larger than that of technological regression, indicating that the level of TE is a key way to promote the growth of grain enterprises’ TFP. The more important method is to optimize the factor allocation of grain processing enterprises. Third, differences in TE between regions directly lead to differences in grain enterprises’ TFP and the TE of grain enterprises is in the range of 0.8418 to 0.8890 in the sample period. The TE level in the central region is the highest, followed by the eastern region, and the level is lowest in the western region. Fourth, the difference in the TFP growth rate of grain enterprises among different enterprises in the eastern and western regions is gradually narrowing while that of the central one is gradually expanding and there is an obvious technological catch-up effect between and within the regions, especially in the central one. Specific to the 30 provinces, it can be seen that the TFP growth of grain enterprises in each province is negatively correlated and the degree of spatial correlation gradually increases with time.

Further discussion is presented as follows. First, the sample size may be insufficient, as we selected a sample of 245 leading enterprises among the national key leading agricultural ones. It should be noted that these are examples with strong production capacity and the judgement on the trend of the TFP of Chinese grain enterprises may be somewhat biased. Second, there is a lack of comparison of the efficiency of the main grain-producing areas; this paper divides the regions into eastern, central, and western regions according to the traditional division method, and does not divide the main grain-producing areas. The main reason is that the distribution of grain enterprises is too concentrated, which leads to a lack of necessary research samples in some main grain-producing areas. Third, this paper focuses on the comparison of TFP growth rates, which means that more consideration is given to the relative value of TFP changes; we did not analyse and compare the absolute value of grain enterprises’ TFP, which is different from previous research that uses more data from the China Statistical Yearbook. We used the sample of grain enterprises as an emphasis point sample, which allowed us to analyse China’s agricultural TFP from another level and provide other evidence. Of course, this is also the direction of any future improvements of the paper.

We propose the following policy implications. First, governments in various regions of China must focus on improving the grain enterprises’ TFP in formulating relevant policies and reflect the differences, give full play to the specialties of grain production and processing in different regions, and strive to improve the overall grain processing capacity. Second, improving TE is an important way to promote the growth of the TFP of grain enterprises, and there is great room and potential for improving the TE of grain enterprises at this stage; therefore, to further optimize the allocation of enterprise factors, in addition to the investment of enterprise capital and labour, the more important element is the input of agricultural raw materials, especially to improve the substitution elasticity of factors such as capital, labour, and raw materials. Third, governments should strengthen intra-regional technical cooperation and exchanges, thereby promoting the flow of technical elements across regions and reducing differences between and within regions.

## Figures and Tables

**Table 1:** Descriptive statistics analysis.

Variable	Mean	Standard Deviation	Min	Max
Sales income	11.43	1.43	8.70	17.67
Total assets	11.19	1.34	8.94	17.81
Labour input	6.52	1.41	3.33	11.71
Raw material input	11.89	1.49	9.16	18.71

Note: The unit of sales income, total assets, and raw material input of the enterprise are all ten thousand yuan, the unit of labour input is person, and the logarithm has been taken.

**Table 2:** Grain enterprise meta-frontier TFP index and decomposition from 2014–2017.

Region	Year	MMI	MEC	MTC
China	2014–2015	0.9969	1.0184	0.9791
	2015–2016	1.0412	0.9586	1.0867
	2016–2017	0.9973	0.9979	0.9995
	Mean	1.0118	0.9916	1.0218
Eastern	2014–2015	0.9956	1.0149	0.9811
	2015–2016	1.0367	0.9608	1.0798
	2016–2017	0.9947	0.9958	0.9989
	Mean	1.0090	0.9905	1.0199
Central	2014–2015	0.9981	1.0196	0.9793
	2015–2016	1.0454	0.9585	1.0910
	2016–2017	0.9987	0.9989	0.9999
	Mean	1.0141	0.9923	1.0234
Western	2014–2015	0.9964	1.0204	0.9768
	2015–2016	1.0397	0.9564	1.0878
	2016–2017	0.9983	0.9988	0.9995
	Mean	1.0115	0.9918	1.0214

Note: The mean is the result calculated by the arithmetic mean, the same below.

**Table 3:** Group decomposition of grain enterprises’ TFP from 2014–2017.

Region	Year	MEC		MTC	
		GEC	PTCU	GTC	PTRC
China	2014–2015	1.0039	1.0150	0.9917	0.9879
	2015–2016	0.9693	0.9905	1.0748	1.0118
	2016–2017	0.9978	1.0005	0.9998	0.9997
	Mean	0.9903	1.0020	1.0221	0.9998
Eastern	2014–2015	1.0084	1.0071	0.9880	0.9938
	2015–2016	0.9548	1.0074	1.0955	0.9864
	2016–2017	0.9973	0.9989	0.9985	1.0005
	Mean	0.9868	1.0045	1.0273	0.9936
Central	2014–2015	1.0010	1.0192	0.9934	0.9865
	2015–2016	0.9809	0.9793	1.0599	1.0299
	2016–2017	0.9974	1.0019	1.0008	0.9991
	Mean	0.9931	1.0002	1.0180	1.0052
Western	2014–2015	1.0031	1.0175	0.9933	0.9837
	2015–2016	0.9679	0.9885	1.0746	1.0127
	2016–2017	0.9989	1.0001	0.9997	0.9998
	Mean	0.9900	1.0020	1.0225	0.9987

**Table 4:** Grain enterprises’ technology efficiency of group frontier and meta-frontier from 2014–2017.

Year	GTE				MTE			
	China	Eastern	Central	Western	China	Eastern	Central	Western
2014	0.9042	0.8949	0.8936	0.9303	0.8692	0.8694	0.8726	0.8636
2015	0.9073	0.9020	0.8940	0.9330	0.8846	0.8818	0.8890	0.8810
2016	0.8793	0.8615	0.8763	0.9034	0.8482	0.8473	0.8523	0.8430
2017	0.8772	0.8589	0.8740	0.9022	0.8462	0.8436	0.8511	0.8418
Mean	0.8920	0.8793	0.8845	0.9172	0.8597	0.8605	0.8663	0.8573

**Table 5:** Test results of TFP growth convergence in grain enterprises.

Model	Region	σ/β	P	C	Conclusion
Model 1 (σ convergence)	China	0.0000	0.731	0.0715	not significant
	Eastern	−0.0036 ***	0.000	7.1999	convergence
	Central	0.0029 ***	0.000	−5.8819	divergence
	Western	−0.0006 ***	0.009	1.2031	convergence
Model 2 (absolute β convergence)	China	−1.1800 ***	0.000	0.0133	convergence
	Eastern	−1.1252 ***	0.000	0.0094	convergence
	Central	−1.1980 ***	0.000	0.0160	convergence
	Western	−1.2415 ***	0.000	0.0139	convergence
Model 3 (conditional β convergence)	China	−1.9499 ***	0.000	0.0358	convergence
	Eastern	−1.9010 ***	0.000	0.0289	convergence
	Central	−1.9951 ***	0.000	0.0416	convergence
	Western	−1.9003 ***	0.000	0.0342	convergence

Note: *** is significant at the 1% level.

**Table 6:** Moran’s I of grain enterprises’ TFP.

Year	I	Z	P
2015	−0.171	−1.129	0.130
2016	−0.212	−1.459	0.072 *
2017	−0.270	−1.935	0.026 **

Note: ** and * are significant at the 5% and 10% levels, respectively.

## Data Availability

The data presented in this study are available on request from the corresponding author.
